# Roxadustat alleviates the inflammatory status in patients receiving maintenance hemodialysis with erythropoiesis-stimulating agent resistance by increasing the short-chain fatty acids producing gut bacteria

**DOI:** 10.1186/s40001-023-01179-3

**Published:** 2023-07-10

**Authors:** Xiu-Nan Zhao, Shu-Xin Liu, Zhen-Zhen Wang, Shuang Zhang, Lian-Lian You

**Affiliations:** 1 Department of Nephrology, Dalian Municipal Central Hospital, No. 826, Xinan Road, Dalian, 116033 Liaoning China; 2 Dalian Key Laboratory of Intelligent Blood Purification, Dalian Municipal Central Hospital, No. 826, Xinan Road, Dalian, 116033 Liaoning China; 3 School of Clinical Medicine, Faculty of Medicine, Dalian University of Technology, No. 2, Linggong Road, Dalian, 116024 Liaoning China

**Keywords:** Gut microbiota, Hemodialysis, Renal anemia, Erythropoiesis-stimulating agent, Drug resistance, Roxadustat

## Abstract

**Background:**

Hypoxia-inducible factor-prolyl hydroxylase inhibitors (HIF-PHIs) have improved the treatment of renal anemia, especially in patients resistant to erythropoiesis-stimulating agents (ESAs). HIF facilitates maintain gut microbiota homeostasis, which plays an important role in inflammation and iron metabolism, which are in turn key factors affecting ESA resistance. The current study aimed to investigate the effects of roxadustat on inflammation and iron metabolism and on the gut microbiota in patients with ESA resistance.

**Methods:**

We conducted a self-controlled, single-center study including 30 patients with ESA resistance undergoing maintenance hemodialysis. All patients received roxadustat without iron agents for renal anemia. Hemoglobin and inflammatory factors were monitored. Fecal samples were collected before and after 3 months’ administration and the gut microbiota were analyzed by 16S ribosomal RNA gene sequencing.

**Results:**

Hemoglobin levels increased after treatment with roxadustat for 3 months (*P* < 0.05). Gut microbiota diversity and abundance also changed, with increases in short-chain fatty acid (SCFA)-producing bacteria (*Acidaminococcaceae*, Butyricicoccus, *Ruminococcus bicirculans*, *Ruminococcus bromii*, *Bifidobacterium dentium*, *Eubacterium hallii*) (*P* < 0.05). Serum SCFA levels also increased (*P* < 0.05). Inflammatory factors, including interleukin (IL)-1, IL-6, tumor necrosis factor (TNF)-α, interferon-γ, and endotoxin gradually decreased (*P* < 0.05). Serum hepcidin, ferritin, and total and unsaturated iron-binding capacities decreased (*P* < 0.05), while soluble transferrin receptor levels increased at each time point (*P* < 0.05). There were no significant differences in serum iron and transferrin saturation at each time point. The abundance of *Alistipes shahii* was significantly negatively correlated with IL-6 and TNF-α (*P* < 0.05).

**Conclusions:**

Roxadustat alleviated renal anemia in patients with ESA resistance by decreasing inflammatory factors and hepcidin levels and improving iron utilization. These effects were at least partly mediated by improved diversity and abundance of SCFA-producing gut bacteria, probably via activation of HIF.

## Introduction

Anemia is a common complication of chronic kidney disease (CKD). Renal anemia is estimated to affect 2 to 4 million of the approximately 20 million people in the United States with CKD [1], and > 90% of the 500,000 patients who undergo dialysis in China, leading to high hospitalization rates and a high incidence of cardiovascular events [2]. Renal anemia is a multifactorial process caused by relative erythropoietin (EPO) deficiency, uremia-induced inhibitors of erythropoiesis, shortened erythrocyte survival, and disordered iron homeostasis [3]. Resistance to erythropoiesis-stimulating agents (ESAs) is mainly associated with abnormal iron metabolism and inflammatory anemia [4–6]. There has recently been great progress in the development of drugs for the treatment of renal anemia, including hypoxia-inducible factor-prolyl hydroxylase inhibitors (HIF-PHIs), which have been shown to treat renal anemia safely and effectively, via multiple mechanisms [7]. The representative HIF-PHI, Roxadustat (FG-4592, FibroGen, Inc. San Francisco, CA, USA), has been widely used in clinical applications. Roxadustat induces hypoxia in cells, thus stimulating endogenous EPO synthesis, improving iron metabolism, and reducing inflammation [8]. HIF transcription factors comprise two subunits: an oxygen-sensitive α-subunit (HIF-1α, HIF-2α, or HIF-3α) and a constitutively expressed β-subunit [9]. HIF-1α is a master transcription factor related to cell proliferation and survival, iron metabolism, angiogenesis and glucose metabolism [10]. Notably, HIF-1α has been shown to maintain intestinal homeostasis by not only regulating the integrity of the intestinal epithelial barrier, but also improving the survival of intestinal microorganisms [11], while HIF-2α is involved in the regulation of intestinal iron transporters mediated by the intestinal microbiota. Intestinal microorganisms and their metabolites may inhibit HIF-2α under conditions of iron deficiency [12]. As the master intestinal transcriptional regulator of apical and basolateral iron transporters, HIF-2α is also sensitive to cellular iron and oxygen levels, which can affect transcriptional activation of the iron-absorption machinery to maintain systemic iron levels [13–16].

The human gut microbiota is a complex ecosystem and a key component of gastrointestinal homeostasis. Many studies have shown that the gut microbiota in patients with CKD differs compared with that in healthy people [17–19]. Changes in the gut microbiota and host reactions are related to the progression of CKD, as well as the increased risk of cardiovascular disease, uremic toxins, and inflammation [20, 21]. In 2011, Ritz proposed an “intestinal-renal syndrome” and derived the theory of an “intestinal-renal axis”, based on extensive studies [22]. This concluded that there was a bidirectional relationship between the kidney and intestine, in which functional impairment of one side could affect the normal function of the other side through various mechanisms, with important roles for the gut microbiota and its metabolites.

The mechanism of HIF-PHIs in the treatment of renal anemia has not yet been fully elucidated. We therefore conducted a prospective cohort study to explore the effect of roxadustat on the gut microbiota in hemodialysis (HD) patients with renal anemia and ESA resistance. The aim of this study was to determine if roxadustat could improve renal anemia in patients with ESA resistance by altering the intestinal microbiota, thereby correcting inflammation and iron metabolism disorders.

## Materials and methods

### Study population

Patients with renal anemia who underwent HD during January 2020 to December 2020 at Blood Purification Center of Dalian Municipal Central Hospital were enrolled in this study. The inclusion criteria were: patients aged ≥ 18 years with ESA resistance, whose dialysis age is more than 3 months, who had received stable HD three times weekly for > 12 weeks and who had complete medical records. The exclusion criteria were: diarrhea, enteral or parenteral nutrition intervention, gastrointestinal tumors, biliary inflammation, inflammatory bowel disease, and use of antibiotics, immunosuppressants, glucocorticoids, or probiotics in the past 3 months.

Anemia in CKD patients was defined according to the 2004 European Best Practice Guidelines Working Group [23] as follows: hemoglobin (Hb) levels were considered to be below normal if they were < 11.5 g/dL in women and < 13.5 g/dL in men (< 12 g/dL in those aged > 70 years), for patients living at altitudes < 1500 m.

ESA resistance was defined as failure to attain the target Hb concentration (Hb < 11 g/dL) despite receiving > 300 IU/kg/week (20,000 IU/week) erythropoietin or 1.5 mg/kg darbepoetin-alfa (100 mg/week) over a 3-month period, or a continued need for high dosages to maintain the target Hb level [23].

All study protocols conformed to the principles of the Declaration of Helsinki and were approved by the institutional medical ethics committee of Dalian Municipal Central Hospital (No. YN2022-039-10), and all study patients provided written informed consent. The clinical information was extracted from the electronic medical records system of Dalian Municipal Central Hospital, and the privacy of the patients was fully protected. Fecal specimens were collected after routine diagnosis and treatment in the clinic, with no harm to the patients.

### Laboratory measurements

The blood samples before and after 1 month, 3 months the application of roxadustat were collected. Approximately 3–5 mL venous blood samples for routine laboratory determinations were obtained immediately before dialysis treatment. Hemoglobin was determined by colorimetry using blood cell analyzer (XN-10, sysmex, Japan). Inflammatory factors, including interleukin (IL)-1, IL-6, tumor necrosis factor (TNF)-α, interferon (IFN)-γ, endotoxin and EPO were measured by enzyme‐linked immunosorbent assay (ELISA) using the kit of DRG from Germany. Iron-metabolism indicators, including hepcidin, ferritin, total iron-binding capacity (TIBC), unsaturated iron-binding capacity (UIBC), transferrin saturation (TS), serum iron (SI), soluble transferrin receptor (sTfR) and serum short-chain fatty acids (SCFAs) were measured by ELISA using the kit of Meimian company from Jiangsu, China. Other laboratory parameters including albumin, creatine, urea nitrogen, calcium, phosphorus, β2 microglobulin, total cholesterol, and triglycerides were measured using an ADVIA 2400 Automatic Biochemistry Analyzer (Siemens, Germany). Dialysis adequacy was determined by the single-pool Kt/*V* (spKt/*V*), which was calculated quarterly using the second-generation equation of Daugirdas [24] based on pre- and post-HD blood urea, as follows: Kt/*V* = −ln(*R* − 0.008 × *t*) + [(4 − 3.5 × *R*) × UF/*W*], where *R* is the ratio of post-dialysis to pre-dialysis serum urea nitrogen concentration; *t* is the duration of HD (in hours); UF is the amount of ultrafiltration (in liters) during the HD session; and *W* is the post-dialysis weight (in kilograms).

### Study design

This was a before–after control study of patients with renal anemia treated with roxadustat. All patients initially received roxadustat thrice weekly, based on body weight: patients weighing 45–< 60 kg received 100 mg roxadustat, and those weighing ≥ 60 kg received 120 mg three times weekly. No participant received iron agents during the study. Fecal specimens were collected before treatment and 3 months after the initiation of treatment with roxadustat. During the treatment, the hemoglobin level was measured every 2 weeks to adjust the dose of Roxadustat so that the hemoglobin level of participants can achieve the target of 110–120 g/L. Changes in Hb concentration, levels of inflammatory factors, indices of iron metabolism, and the occurrence of adverse effects were examined during the 3-month treatment period. The above information was obtained from medical records. Group A refers to the patients undergoing maintenance HD before treatment with roxadustat, while Group B refers to the same patients after treatment with roxadustat for 3 months.

### Sample collection, DNA extraction, and 16S rRNA gene amplification sequencing

Fecal samples were collected at the hospital before each chemotherapy. Fresh fecal samples were collected from enrolled patients after natural defection in the clean toilet, using the scoop in the collection tube take 3–5 g fecal and place in the collection tube. The microbial genome was extracted using QIAamp Fast DNA Stool Mini Kit (Qiagen, Hilden, Germany) according to the manufacturer’s instructions. Sample DNA purity and concentration were tested using a Nanodrop 2000 Spectrophotometer. We amplified the bacterial 16S ribosomal RNA gene V3–V4 region using the TransGen AP221-02 Kit (TransGen, Beijing, China). The following PCR primers were used: 338F 5′-ACTCCTACGGGAGGCAGCAG-3′ and 806R 5′-GGACTACHVGGGTWTCTAAT-3′. The reaction volume (20 μL) comprised 5× FastPfu Buffer (4 μL), 2.5 mM dNTPs (2 μL), forward primer (0.8 μL), 5 μM reverse primer (0.8 μL), FastPfu Polymerase (0.4 μL), and template DNA (10 ng). Cycling proceeded as follows: 3 min at 95 °C twenty-seven cycles (30 sat 95 °C, 30 sat 55 °C, 45 sat 72 °C); 10 min at 72 °C. After amplicons extraction, samples were purified and quantified using the AxyPrep DNA Gel Extraction Kit (Axygen Biosciences, CA, USA) and QuantiFluor™-ST (Promega, Madison, WI, USA), respectively. Purified amplicons were pooled in equimolar proportions and paired-end sequenced (2 × 250 bp) on the Illumina MiSeq platform with TruSeqTM DNA Sample Prep Kit (Illumina, San Diego, CA, USA).

### Bioinformatics

All fecal samples were analyzed by Novogene Co. Ltd. (Beijing, China). Paired-end sequencing of the samples was then carried out using the Illumina NovaSeq sequencing platform. After read splicing and filtering, clustering of operational taxonomic unit (OTUs), species annotation, and abundance analysis were carried out using alpha and beta diversity analyses to examine the diversity of the gut microbiota. Alpha diversities of the fecal flora before and after roxadustat treatment were accessed using five metrics: Venn diagrams, species accumulation boxplots, rank abundance curves, rarefaction curves, and Shannon plots, which show species enrichment and distribution. Beta diversity is used to assess variations in species diversity between different environments. PCoA and PCA analyses [25, 26] showed the total intestinal flora differences. MRPP analysis showed differences in the bacterial compositions of the microbiota between two groups. T-tests provided the differences in the gut microbiota at each classification level (phylum, class, order, family, genus, species) between the two groups which emphasizes statistical significance and biological correlation, which can be used to identify significant differences in biomarkers between groups.

### Sample size calculation

The equation for calculating sample size [27] is shown below.

$$n = \frac{{z^2 \times \hat{p}\left( {1 - \hat{p}} \right)}}{\varepsilon^2 },$$

*z* is the *z* score, *ε* is the margin of error, *n* is the population size, *p̂* is the population proportion.

Determine the sample size necessary to estimate the alteration of gut microbiota after the application of roxadustat that identify as vegan with 95% confidence, and a margin of error of 20%. Assume a population proportion of 0.5, and unlimited population size. Remember that *z* for a 95% confidence level is 1.96. Refer to the table provided in the confidence level section for *z* scores of a range of confidence levels.

$$n = \frac{1.96^2 \times 0.5 \times 0.5}{{0.2^2 }} = 24.01.$$

Thus, for the case above, a sample size of at least 24 participants would be necessary.

### Statistical analysis

Continuous normally distributed variables were expressed as mean ± standard deviation and categorical variables were expressed as numbers and percentages. Data before and after the application of roxadustat at each time point were assessed by repeated-measures ANOVA, using Bonferroni analysis. The relationships between gut microbiota and clinical parameters were analyzed by Spearman’s correlation coefficient. Statistical analysis was performed using SPSS, version 23 (IBM Corp., USA). Significant differences in relative abundances of genus between groups were corrected by Benn-Hochberg False discovery rate (FDR). All statistical tests were 2-sided, and *P*-values < 0.05 were considered statistically significant. To detect the alteration in the inflammatory status after the application of Roxadustat, a sample size of 32 patients per group was necessary with a two-sided 5% significance level and a power of 80%.

## Results

**Fig. 1:**
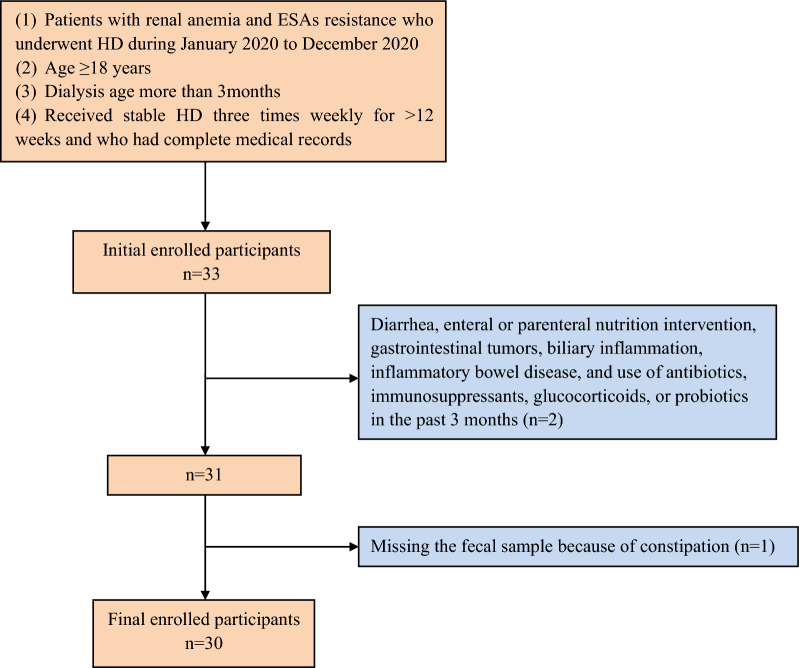
Participants inclusion chart

**Fig. 2:**
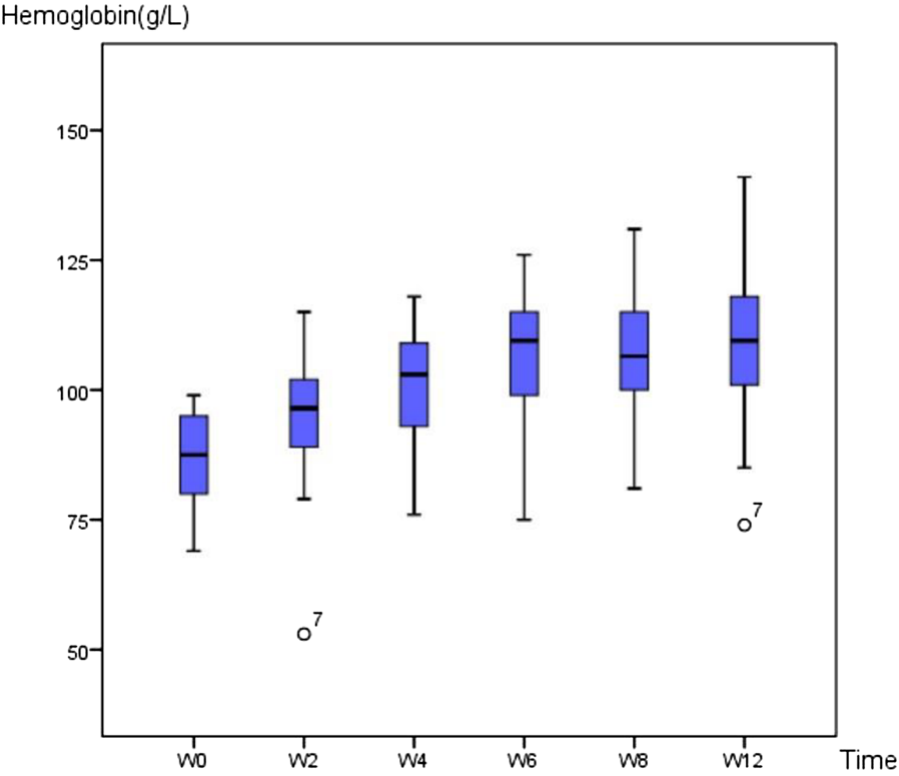
Changes of hemoglobin concentration. W: weeks since application of roxadustat

A total of 30 patients were included in this study to compare the changes in the gut microbiota spectrum after 3 months of roxadustat treatment (Fig. 1). The baseline characteristics of the participants are shown in Table 1. All participants were of Han ethnicity, born in northeastern China, and had a similar dietary pattern which did not alter during the 3 months. No adverse effects were observed during the 3 months of the participants’ medication. Changes in Hb levels during the 3 months are shown in Fig. 2. The Hb level in patient 7 was an outlier, but the results were confirmed by repeated analysis. Baseline Hb at enrollment was (85.93 ± 9.85) g/L, and this increased significantly to (94.97 ± 12.14) g/L after 2 weeks’ of roxadustat treatment (*P* < 0.001). Hb levels continued to increase gradually to (100.80 ± 11.97) g/L, (106.57 ± 13.06) g/L, and (109.20 ± 14.78) g/L after the 1st, 2nd, and 3rd months, respectively (*P* < 0.001). Ferritin, TIBC, UIBC, and hepcidin decreased significantly after the 1st and 3rd months (*P* < 0.05), while sTfR increased significantly (*P* < 0.05). Changes in iron-metabolism indices are shown in Table 2. Levels of inflammatory factors decreased significantly after the 1st and 3rd months (*P* < 0.001), as shown in Table 3.

**Table 1:** Baseline characteristics of the participants

Variables	Values
Age (year)	54.40 ± 17.08
Gender (male%)	56.67
Dialysis age (year)	4.07 ± 3.87
Height (m)	1.69 ± 0.08
Weight (kg)	71.91 ± 16.76
Body mass index (kg/m^2^)	24.83 ± 5.10
Diabetes mellitus (%)	40.00
Coronary heart disease (%)	16.67
Hemoglobin (g/L)	89.27 ± 10.77
Albumin (g/L)	39.20 ± 5.07
Creatine (μmol/L)	839.87 ± 264.70
Urea nitrogen (mmol/L)	28.37 ± 6.79
Calcium (mmol/L)	2.20 ± 0.18
Phosphorus (mmol/L)	2.04 ± 0.58
β2 microglobulin (mmol/L)	41.83 ± 13.15
Triglyceride (mmol/L)	2.80 ± 2.62
Cholesterol (mmol/L)	3.69 ± 1.00
Kt/*v*	1.25 ± 0.20

Data are presented as means and standard deviations for continuous variables and as frequencies and percentages for categorical variables

**Table 2:** Changes in iron-metabolism indices after initiation of roxadustat

Time	Ferritin (ng/mL)	SI (umol/L)	TIBC (umol/L)	UIBC (umol/L)	TS (%)	STfR (mg/L)	Hepcidin (ng/L)
Baseline (M0)	72.96 ± 14.16	15.72 ± 6.21	56.70 ± 10.83	41.49 ± 14.00	27.85 ± 12.20	38.46 ± 7.75	116.12 ± 17.46
M1	65.69 ± 12.00	16.63 ± 7.64	54.59 ± 8.65	37.97 ± 12.35	31.62 ± 15.82	41.63 ± 7.57	86.72 ± 16.97
M3	55.54 ± 13.73	15.47 ± 5.23	48.93 ± 12.97	33.46 ± 13.92	33.58 ± 14.99	50.18 ± 7.73	78.86 ± 15.90
*F* value	10.73	0.489	5.906	3.721	1.582	18.68	1034.523
*P* value	< 0.001	0.602	0.005	0.032	0.218	< 0.001	< 0.001

M0: before roxadustat; M1: 1 month after initiation of roxadustat; M3: 3 months after initiation of roxadustat (end of study)

**Table 3:** Changes in inflammatory factors after initiation of roxadustat

Time	IL-1 (ng/L)	IL-6 (ng/L)	TNF-α (ng/L)	IFN-γ (ng/L)	ET (ng/L)
Baseline (M0)	107.72 ± 17.54	36.54 ± 5.84	55.13 ± 10.58	52.28 ± 8.94	7.82 ± 1.94
M1	85.13 ± 15.97	30.05 ± 5.41	44.14 ± 10.02	37.15 ± 9.11	6.11 ± 1.72
M3	73.75 ± 16.26	28.84 ± 5.68	42.97 ± 9.93	35.42 ± 8.70	5.54 ± 1.55
*F* value	603.626	271.113	321.807	906.224	493.483
*P* value	< 0.001	< 0.001	< 0.001	< 0.001	< 0.001

M0: before roxadustat; M1: 1 month after initiation of roxadustat; M3: 3 months after initiation of roxadustat (end of study)

EPO levels increased significantly (*F* = 1220.882, *P* < 0.001) from (6.76 ± 1.69) U/L at baseline to (9.33 ± 1.62) U/L after 1 month and (10.13 ± 1.56) U/L after 3 months.

Serum SCFA levels also increased significantly (*F* = 23.577, *P* < 0.001) from (53.21 ± 13.72) g/L at baseline to (60.71 ± 12.36) g/L after 1 month and (69.73 ± 11.67) g/L after 3 months.

### Species richness and diversity

**Fig. 3:**
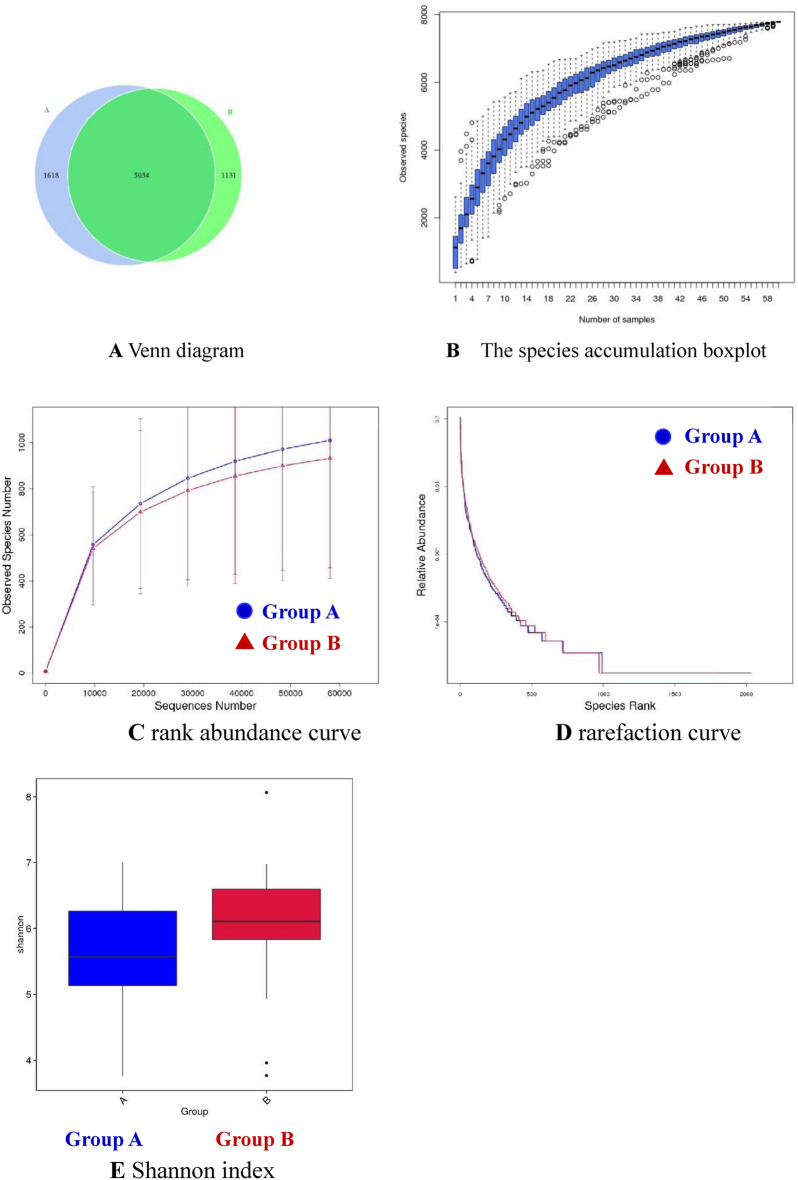
**A** Venn diagram. **B** The species accumulation boxplot. **C** Rank abundance curve. **D** Rarefaction curve. **E** Shannon index

We assessed the alpha diversities of the fecal flora before and after roxadustat treatment using five metrics: Venn diagrams, species accumulation boxplots, rank abundance curves, rarefaction curves, and Shannon plots, to show species enrichment and distribution. Following clustering, common and unique OTUs among the groups were analyzed and displayed in a Venn diagram. There were 1618 unique OTUs in Group A, 1131 unique OTUs in Group B, and 5034 OTUs that were shared by both groups (Fig. 3A). The species accumulation boxplot suggests that the boxplot tends toward a more gradual position, indicating that the sample size was sufficient for analysis of the fecal microbiota (Fig. 3B). The slope of the rank abundance curve reflected the richness and evenness of the species in the sample (Fig. 3C): species richness was reflected by the width of the curve, and the uniformity of species in the sample was reflected by the smoothness of the curve in the vertical direction. The rarefaction curve reflected the richness and evenness of the species in the two groups (Fig. 3D): a flat curve indicated that the volume of sequencing data was reasonable. Microbial community diversity and richness were also evaluated using the Shannon indice. According to the OTU distribution, the Shannon index differed significantly between the two groups (Fig. 3E) (*P* = 0.047), suggesting that the community richness and diversity of the two groups differed.

### Differences in microbiota between Group A and Group B

**Fig. 4:**
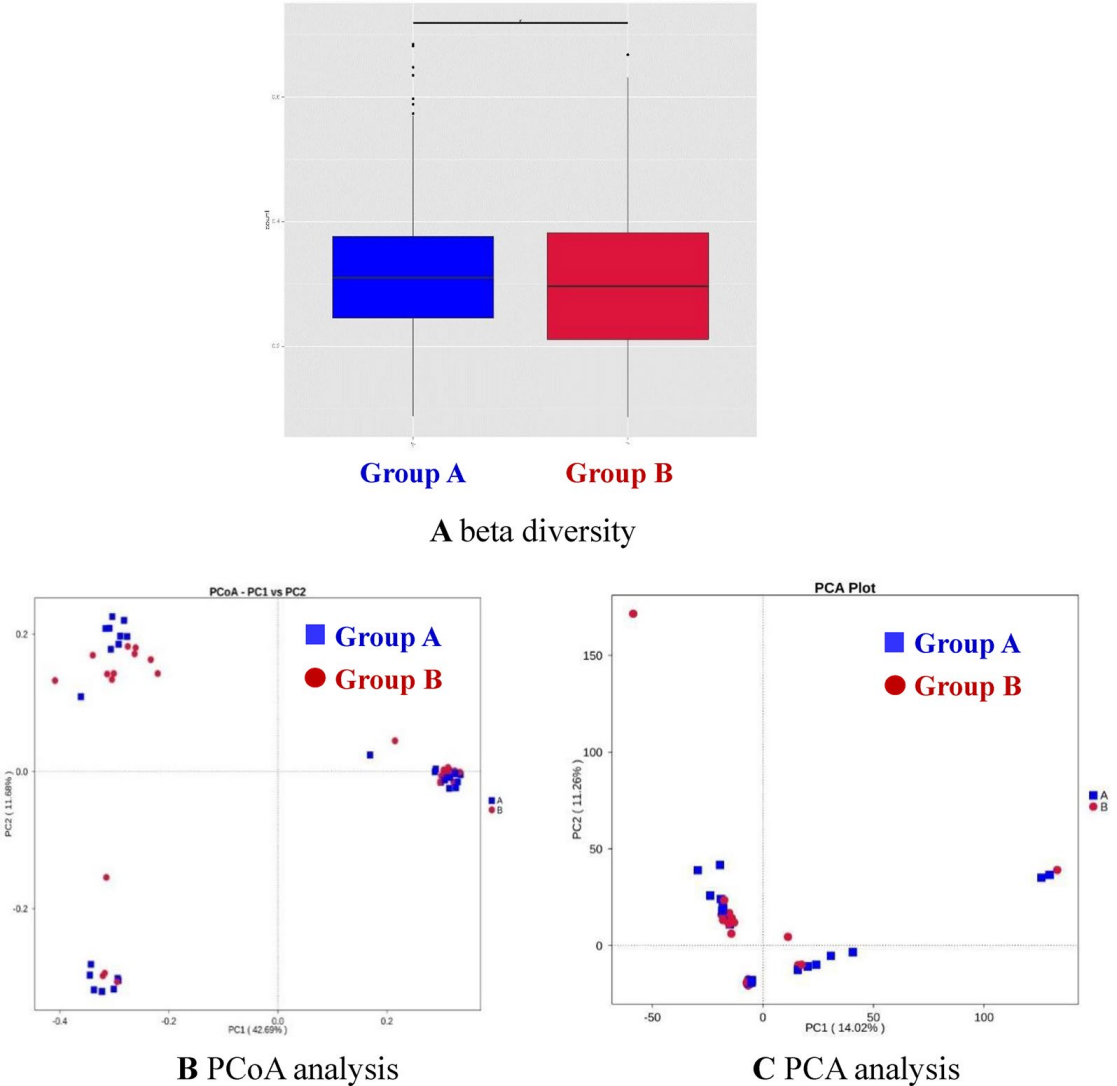
**A** beta diversity. **B** PCoA analysis. **C** PCA analysis

Beta diversity is used to assess variations in species diversity between different environments. The beta diversity measurements of the samples taken before and after roxadustat treatment are shown in Fig. 4A. PCoA (Fig. 4B) and PCA analyses (Fig. 4C) showed that the intestinal flora differed significantly between the two groups, but could not be completely separated. In addition, MRPP analysis (Table 4) also showed significant differences in the bacterial compositions of the microbiota between Groups A and B (*P* = 0.029).

**Table 4:** MRPP analysis between the groups

Group	*A*	Observed-delta	Expected-delta	Significance
A–B	0.006361	0.6359	0.64	0.029

The higher the Observe delta value, the smaller the intra group difference; the higher the Expect Delta value, the greater the inter group difference. *A* value greater than 0 indicates that the difference between groups is greater than the intra group difference, while *A* value less than 0 indicates that the intra group difference is greater than the inter group difference. Significance value less than 0.05 indicates significant difference

**Fig. 5:**
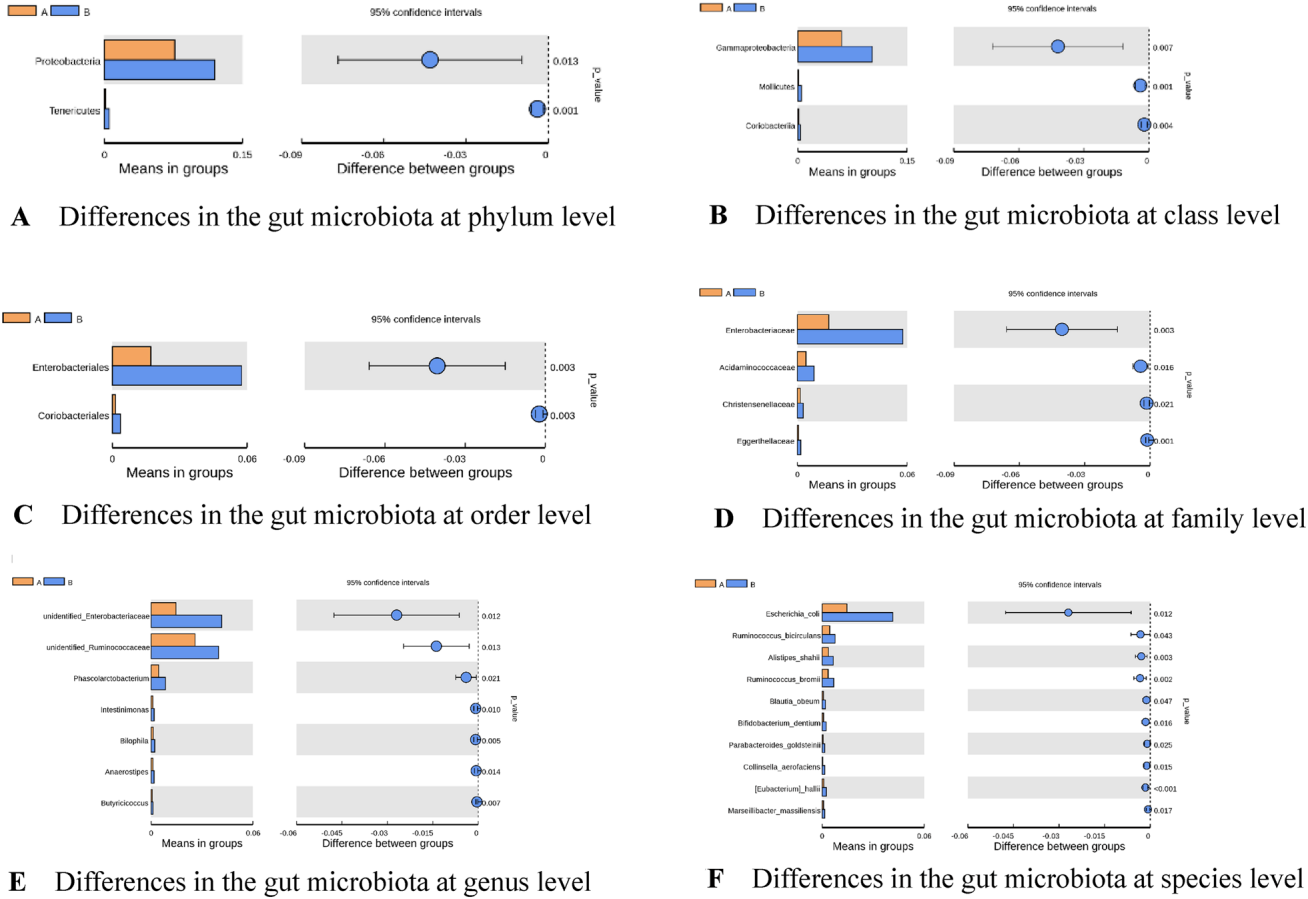
**A** Differences in the gut microbiota at phylum level. **B** Differences in the gut microbiota at class level. **C** Differences in the gut microbiota at order level. **D** Differences in the gut microbiota at family level. **E** Differences in the gut microbiota at genus level. **F** Differences in the gut microbiota at species level

There were significant differences in the gut microbiota at each classification level (phylum, class, order, family, genus, species) between Groups A and B (*t*-tests, *P* < 0.05) (Fig. 5 and Table 5). There were significant differences between the two groups in the abundances of the following: *Proteobacteria* and *Tenericutes* at the phylum level; *Gammaprotobacteria*, *Mollicutes* and *Coriobacteria* at the class level; *Enterobacteriales* and Coriobacteriales at the order level; *Enterobacteriaceae*, *Acidaminococcaceae*, *Christensenellaceae*, and *Eggerthellaceae* at the family level; *unidentified Enterobacteriaceae*, *unidentified Ruminococcaceae*, *Phascolarctobacterium*, *Intestinimonas*, *Bilophia*, *Anaerostipes*, *Butyricicoccus* at the genus level; and *Escherichia coli*, *Ruminococcus bicirculans*, *Alistipes shahii*, *Ruminococcus bromii*, *Blautia obeum*, *Bifidobacterium dentium*, *Parabacteroides goldsteinii*, *Collinsella aerofaciens*, *Eubacterium hallii*, and *Marseillibacter massiliensis* at the species level (all *P* < 0.05). All the above bacteria were more abundant in Group B compared with Group A.

**Table 5:** Differences in the gut microbiota between Groups A and B (*t*-tests)

Bacteria	Abundance in Group A	Abundance in Group B	*P* value	95% CI
*Proteobacteria*	(0.0765 ± 0.041)	(0.1196 ± 0.0814)	0.001	(− 0.0062; − 0.0017)
*Tenericutes*	(0.0013 ± 0.0014)	(0.0052 ± 0.0059)	0.012	(− 0.0767; − 0.0096)
*Gammaprotobacteria*	(0.0601 ± 0.037)	(0.1021 ± 0.0728)	0.007	(− 0.0721; − 0.0119)
*Mollicutes*	(0.0013 ± 0.0014)	(0.0052 ± 0.0059)	0.012	(− 0.0062; − 0.0017)
*Coriobacteria*	(0.0015 ± 0.0013)	(0.0037 ± 0.0036)	0.004	(− 0.0036; − 0.0007)
*Enterobacteriales*	(0.0171 ± 0.0246)	(0.0576 ± 0.0643)	0.003	(− 0.0659; − 0.0150)
Coriobacteriales	(0.0014 ± 0.0013)	(0.0036 ± 0.0036)	0.003	(− 0.0036; − 0.0008)
*Enterobacteriaceae*,	(− 0.0171 ± 0.024)	(0.0576 ± 0.0643)	0.003	(− 0.6592; − 0.0150)
*Acidaminococcaceae*	(0.0047 ± 0.0049)	(0.0090 ± 0.0081)	0.016	(− 0.0078; − 0.0008)
*Christensenellaceae*	(0.0016 ± 0.0017)	(0.0031 ± 0.0003)	0.02	(− 0.0028; − 0.0002)
*Eggerthellaceae*	(0.0006 ± 0.0005)	(0.0018 ± 0.0017)	0.0007	(− 0.0019; − 0.0006)
Unidentified *Enterobacteriaceae*	(0.0146 ± 0.0233)	(0.0416 ± 0.051)	0.012	(− 0.0476; − 0.0062)
Unidentified *Ruminococcaceae*	(0.0259 ± 0.0142)	(0.0398 ± 0.0258)	0.014	(− 0.0247; − 0.0030)
*Phascolarctobacterium*	(0.0045 ± 0.0048)	(0.0084 ± 0.0078)	0.021	(− 0.0074; − 0.0006)
*Intestinimonas*	(0.0009 ± 0.0008)	(0.0178 ± 0.0015)	0.0098	(− 0.0014; − 0.0002)
*Bilophia*	(0.0012 ± 0.0007)	(0.0021 ± 0.0014)	0.0047	(− 0.0015; − 0.0003)
*Anaerostipes*	(0.0011 ± 0.0007)	(0.0017 ± 0.0013)	0.014	(− 0.0012; − 0.0001)
*Butyricicoccus*	(0.0006 ± 0.0003)	(0.0011 ± 0.0008)	0.007	(− 0.0008; − 0.0001)
*Escherichia coli*	(0.0146 ± 0.0233)	(0.0416 ± 0.0510)	0.011	(− 0.0476; − 0.0063)
*Ruminococcus bicirculans*	(0.0045 ± 0.0054)	(0.0077 ± 0.0065)	0.043	(− 0.0063; − 0.0001)
*Alistipes shahii*	(0.0037 ± 0.0036)	(0.0066 ± 0.0038)	0.003	(− 0.0048; − 0.001)
*Ruminococcus bromii*	(0.0035 ± 0.0025)	(0.0068 ± 0.0049)	0.002	(− 0.0054; − 0.001)
*Blautia obeum*	(0.0008 ± 0.0007)	(0.0020 ± 0.0032)	0.047	(− 0.0024; − 0.00002)
*Bifidobacterium dentium*	(0.00006 ± 0.00009)	(0.0002 ± 0.0004)	0.034	(− 0.0031; − 0.00001)
*Parabacteroides goldsteinii*	(0.0006 ± 0.0006)	(0.0016 ± 0.0022)	0.025	(− 0.0018; − 0.0001)
*Collinsella aerofaciens*	(0.0004 ± 0.0008)	(0.0016 ± 0.0022)	0.015	(− 0.002; − 0.0002)
*Eubacterium hallii*	(0.0009 ± 0.0006)	(0.0025 ± 0.0019)	0.0002	(− 0.0023; − 0.0008)
*Marseillibacter massiliensis*	(0.0008 ± 0.0011)	(0.0016 ± 0.0011)	0.017	(− 0.0013; − 0.0001)

**Fig. 6:**
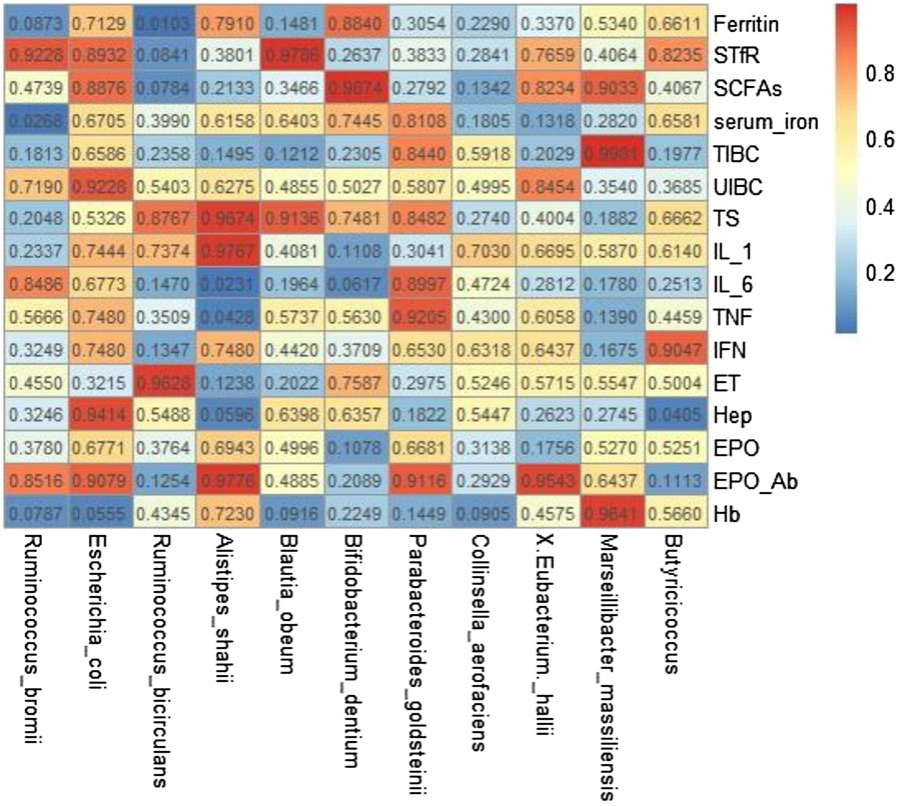
Correlation heatmap

In addition, we investigated the relationships between the alteration of gut microbiota and the *D*-value of clinical parameters before and after the 3 months application of roxadustat including Hb, inflammatory factors, and iron-metabolism indices, using Spearman’s correlation coefficient analysis (Table 6). The correlation heatmap figure is presented in Fig. 6. The abundance of *Alistipes shahii* was significantly negatively correlated with IL-6 (*R* = − 0.41371) and TNF-α (*R* = − 0.37232) (both *P* < 0.05).

**Table 6:** Correlations between gut microbiota and clinical parameters

	Escherichia_coli		Ruminococcus_bicirculans		Alistipes_shahii	
	*R*	*P*	*R*	*P*	*R*	*P*
Ferritin	− 0.07008	0.7129	− 0.46118	0.0103	− 0.0505	0.791
STfR	0.02558	0.8932	− 0.32058	0.0841	− 0.16618	0.3801
SCFAs	0.02694	0.8876	− 0.32636	0.0784	− 0.23397	0.2133
Serum iron	0.081	0.6705	− 0.15977	0.399	− 0.09546	0.6158
TIBC	− 0.08411	0.6586	− 0.22319	0.2358	− 0.26969	0.1495
UIBC	− 0.01846	0.9228	− 0.11635	0.5403	− 0.09232	0.6275
TS	0.11858	0.5326	0.02959	0.8767	− 0.00779	0.9674
IL-1	0.0621	0.7444	− 0.06388	0.7374	0.00556	0.9767
IL-6	− 0.07923	0.6773	− 0.27128	0.147	− 0.41371	0.0231
TNF-α	0.0612	0.748	− 0.17648	0.3509	− 0.37232	0.0428
IFN-γ	0.0612	0.748	− 0.27951	0.1347	− 0.0612	0.748
ET	− 0.18734	0.3215	0.0089	0.9628	− 0.28724	0.1238
Hepcidin	− 0.01402	0.9414	0.11394	0.5488	− 0.34784	0.0596
EPO	− 0.07928	0.6771	− 0.16746	0.3764	− 0.07482	0.6943
EPO-Ab	− 0.02206	0.9079	− 0.2861	0.1254	− 0.00535	0.9776
Hb	− 0.3532	0.0555	− 0.14819	0.4345	− 0.06752	0.723

	Blautia_obeum		Bifidobacterium_dentium		Parabacteroides_goldsteinii	
	*R*	*P*	*R*	*P*	*R*	*P*
Ferritin	− 0.27061	0.1481	− 0.02781	0.884	0.19357	0.3054
STfR	0.00512	0.9786	− 0.2107	0.2637	0.16509	0.3833
SCFAs	− 0.17804	0.3466	0.00301	0.9874	− 0.20416	0.2792
Serum iron	− 0.08893	0.6403	− 0.06209	0.7445	− 0.04562	0.8108
TIBC	− 0.28915	0.1212	− 0.22566	0.2305	0.0375	0.844
UIBC	− 0.13241	0.4855	− 0.12727	0.5027	0.10502	0.5807
TS	0.0207	0.9136	0.06119	0.7481	− 0.03649	0.8482
IL-1	0.15674	0.4081	− 0.29716	0.1108	0.1941	0.3041
IL-6	− 0.24265	0.1964	− 0.3452	0.0617	0.02404	0.8997
TNF-α	− 0.10697	0.5737	− 0.10995	0.563	0.01903	0.9205
IFN-γ	− 0.14581	0.442	0.16937	0.3709	0.08558	0.653
ET	− 0.23959	0.2022	− 0.05852	0.7587	− 0.19671	0.2975
Hepcidin	− 0.08905	0.6398	− 0.09014	0.6357	− 0.25028	0.1822
EPO	− 0.1282	0.4996	− 0.29955	0.1078	− 0.08163	0.6681
EPO-Ab	− 0.13151	0.4885	− 0.23621	0.2089	0.02117	0.9116
Hb	− 0.31353	0.0916	0.22833	0.2249	− 0.27268	0.1449

	Collinsella_aerofaciens		Eubacterium_hallii			Marseillibacter_massiliensis
	*R*	*P*	*R*	*P*	*R*	*P*
Ferritin	− 0.22638	0.229	− 0.18156	0.337	0.11816	0.534
STfR	− 0.20211	0.2841	0.05674	0.7659	− 0.15732	0.4064
SCFAs	− 0.27988	0.1342	0.04252	0.8234	− 0.02316	0.9033
Serum iron	− 0.25125	0.1805	− 0.28152	0.1318	− 0.20298	0.282
TIBC	− 0.10197	0.5918	− 0.23923	0.2029	− 0.00045	0.9981
UIBC	0.12821	0.4995	0.03716	0.8454	0.17534	0.354
TS	− 0.20634	0.274	− 0.15931	0.4004	− 0.247	0.1882
IL-1	− 0.0726	0.703	0.08125	0.6695	− 0.10329	0.587
IL-6	− 0.13638	0.4724	− 0.20332	0.2812	− 0.25264	0.178
TNF-α	− 0.14963	0.43	− 0.09815	0.6058	− 0.27657	0.139
IFN-γ	− 0.09118	0.6318	0.08803	0.6437	0.25865	0.1675
ET	− 0.12088	0.5246	− 0.10759	0.5715	0.11227	0.5547
Hepcidin	− 0.11512	0.5447	− 0.21133	0.2623	− 0.20612	0.2745
EPO	− 0.19029	0.3138	− 0.25401	0.1756	− 0.12017	0.527
EPO-Ab	− 0.19853	0.2929	− 0.01092	0.9543	− 0.08803	0.6437
Hb	− 0.31449	0.0905	− 0.14096	0.4575	− 0.00858	0.9641

	Butyricicoccus		Ruminococcus_bromii			
	*R*	*P*	*R*	*P*		
Ferritin	0.11816	0.534	− 0.3175	0.0873		
STfR	− 0.15732	0.4064	0.01847	0.9228		
SCFAs	− 0.02316	0.9033	− 0.13592	0.4739		
Serum iron	− 0.20298	0.282	− 0.40414	0.0268		
TIBC	− 0.00045	0.9981	− 0.25081	0.1813		
UIBC	0.17534	0.354	− 0.25081	0.1813		
TS	− 0.247	0.1882	− 0.23829	0.2048		
IL-1	− 0.10329	0.587	0.22415	0.2337		
IL-6	− 0.25264	0.178	0.22415	0.2337		
TNF-α	− 0.27657	0.139	− 0.10895	0.5666		
IFN-γ	0.25865	0.1675	− 0.10895	0.5666		
ET	0.11227	0.5547	− 0.14174	0.455		
Hepcidin	− 0.20612	0.2745	− 0.14174	0.455		
EPO	− 0.12017	0.527	− 0.14174	0.455		
EPO-Ab	− 0.02206	0.9079	− 0.2861	0.1254		
Hb	− 0.3532	0.0555	− 0.14819	0.4345

## Discussion

The current study showed that Hb and EPO levels were increased after the administration of roxadustat in patients with ESA resistance undergoing maintenance HD. Roxadustat regulated the levels of inflammatory factors, increased iron utilization, and altered the diversity and abundance of the gut microbiota, especially in terms of SCFA-producing bacteria, thus alleviating ESA resistance.

In line with previous studies, roxadustat can effectively treat patients with renal anemia in end-stage renal disease (ESRD) [28, 29]. Numerous clinical trials have shown the advantage of roxadustat compared with epoetin alfa [30]. The mechanism of roxadustat involves stimulating the manufacture of endogenous EPO, improving iron utilization [31] (indicated by suppression of hepatic hepcidin and upregulation of transport genes related to iron metabolism, such as *DCYTB*, *DMT1*, and *TF*), and reducing inflammation. Roxadustat is known to adjust the inflammatory microenvironment and ameliorate disorders of iron metabolism (the two main characteristics of renal anemia) in CKD patients, especially those with ESA resistance [3, 32–35]. Notably, our results showed a dramatic change in the gut microbiota after the administration of roxadustat, particularly characterized by an increase in SCFA-producing bacteria. Meanwhile, our study showed the abundance of *Alistipes shahii* was negatively correlated with IL-6 and TNF-α levels. *Alistipes shahii*, known as SCFAs-producing bacteria, can significantly increase acetate and propionate so that relieve the bowel inflammation [36]. These results thus suggest that roxadustat may alleviate ESA resistance by regulating the gut microbiota.

CKD is considered to be a chronic inflammatory condition [37, 38] and to be closely linked to the gut microbiota [39, 40]. There is growing acceptance that the gut microbiota and its products affect the pathogenesis and development of CKD. Several factors, such as toxin accumulation [41–43], inflammatory status, drugs (corticosteroids, immunosuppressive agents, antibiotics), and dietary restrictions may affect the gut microbiota [44]. Recent studies found differences in the composition and function of the gut microbiota between dialysis patients with ESRD and healthy controls [45–47]. Jiang et al. found that butyrate-producing microbiota, such as *Roseburia* spp. and *Faecalibacterium prausnitzii*, were significantly decreased in patients with any stage of CKD (*n* = 65) compared with healthy controls (*n* = 20) in a study in China. Wong et al. compared patients with ESRD (*n* = 24) and healthy controls (*n* = 12) in the United States, and showed that SCFAs played a dominant role in the gut microbiota related to uremic toxins in ESRD patients. Nineteen microbial families were increased, including 12 with urease-producing enzymes, five with uricase, and four with indole and p-cresol-producing enzymes, while four microbial families, including two with butyrate-producing enzymes, were decreased. Zhao et al. conducted a systematic review [48] of gut microbiota in patients with CKD, which showed significant increases in bacterial families possessing urease, uricase, and indole and p-cresol forming enzymes, and decreases in families possessing butyrate-forming enzymes among ESRD patients [49]. Previous studies comparing the intestinal flora between ESRD patients and healthy controls showed decreases in bacteria producing SCFAs, especially butyrate, in ESRD patients [48, 50]. Notably, however, the current study indicated that SCFA-producing bacteria increased after the administration of roxadustat. We thus speculated that the inflammatory and toxic microenvironment in patients with ESRD may be closely linked to the gut microbiota, which may play a dominant role in regulating ESA resistance.

Our results showed that SCFA-producing bacteria and serum SCFAs were increased and inflammatory factors were decreased after the administration of roxadustat. As an HIF-PHI, roxadustat can activate the HIF/PHD oxygen-sensing pathway, thus inducing the expression of HIF-1α and HIF-2α. HIF-1α has an anti-inflammatory effect while HIF-2α affects iron utilization.

Recent studies have highlighted the prominent role of HIF-1α in maintaining intestinal homeostasis, not only by regulating the integrity of the intestinal epithelial barrier but also improving the survival of intestinal microorganisms [11]. The intestinal lumen in mammals is characterized by a hypoxic environment, which can induce the expression of several HIF-1 target genes in intestinal epithelial cells. Thus HIF-1α up-regulates genes involved in intestinal barrier function, such as *MUC2*, *ITF*, *CLDN1*, and other tight-junction proteins, and down-regulates nuclear factor (NF)-κB signal transduction and the production of anti-inflammatory cytokines to exert an immunosuppressive effect [51]. HIF-1α induced the development of SCFA-producing bacteria, which are key to maintaining intestinal homeostasis. First, SCFAs especially butyrate, provides the energy framework of colon cells [52, 53], also plays a crucial role in maintaining intestinal epithelial integrity, which is related to its ability to produce mucin and tight-junction proteins [54–56]. Second, SCFAs, as a major mode of communication between the microbiota and host cells, have demonstrated several immunomodulatory effects [57]. SCFAs have anti-inflammatory effects by reducing the secretion of proinflammatory cytokines and chemokines, and possibly reducing local macrophage infiltration. SCFAs also exert anti-inflammatory effects through inhibition of NF-κB activation in the host immune cells by binding to G-protein-coupled receptors (GPRs) 43 and 41 [58–60]. In addition, SCFAs epigenetically regulates gene expression by inhibiting histone deacetylases, which can induce the differentiation of colonic Treg cells [61], and can also improve the host response to inflammation [62, 63]. These functions may also be conducive to the survival of the intestinal flora and may promote the interaction between the host and gut microorganisms. Meanwhile, the abundance of *E. coli* increased after Roxadustat treatment. In present, the function of *E. coli* of chronic kidney disease is still inexplicit. There is now substantial research showing that the *E. coli* is more abundant in patients with ESRD [17, 19, 64] and it may produce protein-bound uraemic toxins (PBUTs), such as indoxyl sulfate (IS) and p-cresyl sulfate (PCS) [65]. In vitro, the level of *E. coli* was elevated after the application of rhuEPO. Thus, the upregulation of E. coli after roxadustat treatment maybe a result of the promotion of EPO [66]. The function of *E. coli* in ESRD patients is an unsettled question and need further study.

HIF-1 was first identified as a transcription factor regulating EPO in human hepatoma cells, while in vivo hypoxic stimulation of EPO and erythropoiesis is primarily mediated by HIF-2 [67–69]. Intestinal iron absorption has recently been shown to be mainly regulated by HIF-2α, which is an oxygen- and iron-regulated transcription factor that directly targets the three key intestinal iron transporters: divalent metal transporter 1 (DMT1), duodenal cytochrome b, and ferroportin (FPN) [15]. In the hypoxic environment of the duodenum, HIF-2α upregulates the expression of apical (DMT1) and basolateral (FPN) enterocyte iron transporters, thus improving iron absorption [70]. The importance of hypoxia signaling and the essential role of HIF-2α in iron homeostasis have been highlighted in several genetic and pathophysiologic mouse models [12]. These models showed that the gut microbiota regulates systemic iron homeostasis in the host in two ways: by repressing intestinal iron-absorption pathways via the inhibition of basal HIF-2α function, and by promoting cellular iron storage via the induction of FTN expression. The host iron-sensing mechanism is intimately connected to and regulated by the gut microbiome. The present study showed that hepcidin, ferritin, TIBC, and UIBC all decreased after the administration of roxadustat, indicating improved iron utilization. This could partly explain the reduced demand for iron in patients with renal anemia treated with roxadustat compared with ESAs. Hepcidin is a key regulator of iron absorption [71]. The commensal microbiota could regulate hepcidin expression during intestinal inflammation via STAT3 activation and modulation of erythropoietic activity [72]. Roxadustat could thus improve anemia by affecting iron metabolism, and especially by reducing hepcidin levels [73]. We, therefore, considered that roxadustat might correct ESA resistance via activation of HIF-2α to improve iron utilization.

Our study had several strengths. First, to the best of our knowledge, this was the first study to explore the effects of roxadustat on the gut microbiota in patients with renal anemia and ESA resistance undergoing maintenance HD. The results thus provide a basis for further studies to explore the mechanism of roxadustat for the treatment of renal anemia. Second, this was a before–after control study, which thus eliminated the potential effects of individual differences and other confounding factors on the results, whilst ensuring the accuracy of the results and reducing the sample size, with high statistical efficiency.

The study also had some limitations. First, the limited data meant that the study may have been influenced by some unmeasured confounders, such as exercise, diet, and sleep information. These factors could also affect the gut microbiota [74–76], with potential implications for health. Second, although our results indicated that roxadustat significantly altered the gut microbiota, the species accumulation box plot suggested that the sample size was relatively limited. Further studies with larger sample sizes are therefore required. Third, the study enrolled patients from a single dialysis center, thus limiting the generalizability of the results to the overall HD population. Further studies are, therefore, needed to verify the current results.

## Conclusions

Roxadustat alleviated renal anemia in patients with ESA resistance by decreasing inflammatory factors and hepcidin levels and improving iron utilization. These effects were at least partly mediated by improved diversity and abundance of SCFA-producing gut bacteria, probably via activation of HIF.

## Data Availability

The datasets used and/or analyzed during the current study are available from the corresponding author upon reasonable request.
